# Fixed-time treatment of elderly patients with acute myeloid leukemia (AML) or high-risk myelodysplastic syndrome (MDS) using hypomethylating agents and venetoclax – a case series

**DOI:** 10.1007/s00277-026-07076-z

**Published:** 2026-05-22

**Authors:** Albrecht Lindemann, Gerhard Göckel, Guillermo Garcia-Pardillos

**Affiliations:** Haematology and Oncology Specialist Practice, Wilhelmstraße 1, Ettlingen, D-76275 Germany

**Keywords:** AML, MDS, Hypomethylating agents, Venetoclax

## Abstract

Standard therapy for elderly patients with acute myeloid leukemia (AML) consists of hypomethylating agents (HMAs) combined with venetoclax (VEN), administered long term until disease progression. However, several retrospective studies have shown that patients in complete remission (CR) who discontinued treatment electively or because of poor tolerance did not experience a negative impact on relapse-free or overall survival. Therefore, after obtaining informed consent, we offered treatment discontinuation to patients in CR after six months of HMA-VEN therapy. Here, we report the outcomes of seven consecutively treated patients who achieved CR between May 2021 and June 2025 and opted for discontinuation of therapy. Five of the seven patients showed clearance of somatic mutations to < 1% and resolution of cytogenetic abnormalities. Six of the seven patients remain in continuous remission for 7–50 months. One patient with FLT3-ITD experienced relapse after 14 months and achieved a second hematologic remission after resuming HMA-VEN therapy. These findings suggest that treatment-free remission after time-limited HMA-VEN therapy may be a feasible approach in selected patients.

**Supplementary Information:**

The online version contains supplementary material available at 10.1007/s00277-026-07076-z.

## Introduction

In elderly patients with acute myeloid leukemia (AML) or high-risk myelodysplastic syndrome (MDS), treatment with hypomethylating agents (HMAs) in combination with venetoclax (VEN) has demonstrated encouraging rates of complete remission (CR) and overall survival [1]. Therapy is generally continued until resistance develops or toxicity becomes limiting [2]. However, to date, there are no data demonstrating that continuation of therapy in patients who have achieved CR is necessary or preferable with regard to prognosis and quality of life [3]. Here, we present a case series of seven patients with AML or high-risk MDS, treated between May 2021 and June 2025, in whom treatment was discontinued after six months following achievement of CR.

## Methods

Between May 2021 to June 2025, 19 patients with AML of the elderly or high-risk MDS were treated in our hematology and oncology specialist practice with HMA/VEN. Diagnoses were established according to the International Consensus Classification of Myeloid Neoplasms and Acute Leukemias (ICC 2022) [4]. Risk stratification for AML patients receiving less-intensive therapies was performed in accordance with the 2024 ELN recommendations [5] and for MDS using the Molecular International Prognostic Scoring System for Myelodysplastic Syndromes (IPSS-M) [6].

Bone marrow biopsies were performed to assess morphology, cytogenetics, and somatic mutations by Next-Generation Sequencing (NGS) prior to treatment initiation, in cases of unexplained cytopenia during treatment, and to determine the remission status after 6 months in patients with normalized peripheral blood counts [7, 8].

Molecular genetic analyses were performed using NGS targeting coding regions and relevant flanking intronic sequences with a custom myeloid gene panel (Agilent Technologies), sequenced on Illumina MiSeq or NextSeq platforms. The panel covered recurrently mutated genes in myeloid malignancies (including, among others, ASXL1, DNMT3A, TET2, TP53, RUNX1, SF3B1, SRSF2, U2AF1, NPM1, FLT3, IDH1/2, and RAS pathway genes), with the full list of targeted regions provided in Supplementary Material 1 (hg19 reference genome). Results were reported as Variant Allele Frequency (VAF), defined as the proportion of sequencing reads harboring a given variant, and were assessed at diagnosis and after six months of treatment in patients who had achieved CR.

**Table 1:** Baseline characteristics of the included patients

					Mutations		VAF		
Patient	Sex	Age (years)	ICC 2022	ELN 2024/ IPSS-M	Somatic	Cytogenetics	at diagnosis in %	in CR in %	Time in CR (months)
1	f	88	AML-MR	favorable	IDH2		20	<0.5	50+
					PHF6		16	<0.5	
					STAD2		12	<0.5	
2	f	79	AML-MR	favorable		del 20q12		46XX	45+
3	m	79	AML-MR	favorable	None				23+
4	f	75	AML-MR	intermediate	BCOR		7.4	<0.5	14
					FLT3		2	<0.5	
					IDH2		8.6	<0.5	
					Runx1		7	<0.5	
5	m	81	MDS-EB	very high risk	BCOR		63	<1	13+
					CEBPA		10	<1	
					Runx1		35	<1	
					SRSF2		31	<1	
					STAG2		42	<1	
					Tet2		28	<1	
					TP53 c.820G>		6	<1	
					TP53 del c.653–670		3	28	
						+8		46XY	
6	f	74	AML-MR	favorable	ETV6		29	45	12+
					IDH-2		24	<1	
					PHF		22	<1	
					STAG		18	<1	
7	f	83	AML-MR	intermediate	BCOR		39	<0.5	7+
					BCORL		40	<0.5	
					CSBPA		11	<0.5	
					DNMT3A		89	26	
					NRAS		12	<0.5	
					Runx1		20	<0.5	
					Tet2-c.124del		44	7.1	
					Tet2 c.1843 del		<0.5	4.6	
					Tet2-c.5618T>C		42	6.8	

A “+” after the number of months indicates that the patient was still in remission at the most recent update (12/31/2025). *AML-MR*, acute myeloid leukemia with myelodysplasia-related or myeloproliferative neoplasm-associated features; *CR,* Complete remission; *ELN,* European LeukemiaNet; *f,* female; *ICC,* International Consensus Classification; *IPSS-M,* Molecular International Prognostic Scoring System; *m,* male; *MDS-EB,* myelodysplastic syndrome with excess blasts; *VAF,* Variant allele frequency.

Treatment was initiated upon clinically relevant cytopenia in MDS or at the time of AML diagnosis, except in 2 elderly AML-MR patients, in whom treatment was deferred because of preserved functionally adequate neutrophil and platelet counts.

Therapy consisted of decitabine administered at a dose of 20 mg/m²/day on days 1–5 in outpatients or azacitidine at 75 mg/m2 on days 1–7 in those starting therapy as inpatients, each combined with venetoclax. Venetoclax was administered at 100 mg on day 1, 200 mg on day 2, and 400 mg on days 3–14; however, in cycle 1 it was discontinued on day 7 if neutrophil counts were < 100/ul [9]. Supportive care included filgrastim when neutrophil counts decreased to < 500/ul, and anti-infective prophylaxis was initiated if neutropenia persisted.

After six months of therapy, when peripheral blood counts indicated at least a good partial remission — defined as neutrophils > 1,000/µL, hemoglobin > 10 g/dL, platelet count > 100,000/µL, and blasts 0% — bone marrow biopsy was performed. In cases in which CR was confirmed by < 5% bone marrow blasts [7, 8], treatment discontinuation was offered after patients were informed that continuous therapy represents the current standard of care and provided that written informed consent was obtained. Patients were followed with monthly peripheral blood counts, which were later extended to intervals of every 2–3 months.

The CARE guidelines were applied for reporting of this case series [10].

## Results

Of 19 consecutively treated patients, seven (six with AML-MR and one with high-risk MDS) aged 75–88 years achieved CR after six months of therapy (Tables 1 and 2). All seven patients opted for treatment discontinuation.

Briefly, among the remaining 12 patients, all with AML-MR, 3 patients (aged 61, 66, and 71 years) proceeded to allogeneic transplant: 2 were in CR after 3 and 4 cycles of HMA/VEN, while one patient with biallelic TP53 mutation underwent transplantation after 4 cycles and persistence of 15% bone marrow blasts. 3 patients with MDS/MPN, aged 74, 77 and 82 years, died of progressive disease after 1, 6 and 26 months of therapy, respectively. The remaining 6 patients with MDS associated AML-MR, aged 72–86 years, achieved only temporary disease control lasting 1–12 months. In 2 patients aged 83 and 86 years with > 20% blasts in the peripheral blood, treatment initiation was postponed by 9 and 11 months, respectively, due to preserved neutrophil count and platelet function. Both patients subsequently developed progressive and ultimately fatal disease after the first cycle of HMA/VEN that had been initiated because of critical cytopenia in one patient and AML-related skin infiltration in the other.

**Table 2:** Blood counts of the patients at diagnosis and in complete remission

		Blood counts				
Patient	Time point	Leukocytes/ul	Neutrophils/ul	Platelets/ul	Blasts in PB in %	Blasts in BM in %
1	at diagnosis	2,600	104	99,000	42.0	25.0
	in CR	8,200	5,250	360,000	0.0	<5.0
2	at diagnosis	14,700	5,340	66,000	0.0	50.0
	in CR	3,000	1,420	442,000	0.0	<5.0
3	at diagnosis	80,900	4,000	106,000	1.8	90.0
	in CR	5,300	3,610	137,000	0.0	<5.0
4	at diagnosis	10,500	<10	55,000	83.0	90.0
	in CR	4,900	2,250	218,000	0.0	<5.0
5	at diagnosis	3,500	340	43,000	0.0	8.0
	in CR	6,100	4,620	113,000	0.0	<5.0
6	at diagnosis	1,800	180	97,000	2.6	60.0
	in CR	6,500	3,520	324,000	0.0	<5.0
7	at diagnosis	1,800	70	153,000	13.0	50.0
	in CR	4,100	1,640	221,000	0.0	<5.0

*CR,* Complete remission defined as < 5% marrow blasts and hematologic recovery with neutrophils >1000/ul and platelets > 100.000/ul for AML [7] and MDS [8]; *PB,* Peripheral blood; *BM,* Bone marrow.

The 7 patients who achieved a CR experienced an uneventful treatment course. Patients 1 and 4–7 were treated as outpatients, in patients 2 and 3 treatment was started in the inpatient setting as described in the Methods section. Dose reductions were required in only 2 of these patients (patient 5 and 7) due to prolonged neutropenia. In patient 7, venetoclax was limited to 7 days in cycles II and III, while in patient 5, the treatment-free interval was extended by one and two weeks, respectively. Based on peripheral blood counts, six patients remain in stable remission at the most recent follow-up (December 31, 2025), with a median remission duration of 14 months since treatment discontinuation.

Patient 4, harboring an FLT3-ITD mutation, experienced relapse 14 months after treatment discontinuation. Bone marrow blasts were closely related to the primary clone, sharing the same RUNX1, BCOR, IDH2, and FLT3 mutations, but had acquired a second FLT3-ITD. After two cycles of retreatment with the same decitabine and venetoclax regimen used in first-line therapy, the patient achieved a second hematologic remission, with peripheral blood blasts < 1%, neutrophil counts of 1,160/µL, and platelet counts of 151,000/µL.

Patient 5, diagnosed with MDS-EB [4] and classified as very high risk according to IPSS-M, received off-label HMA/VEN therapy based on the expectation that higher CR rates with combination therapy might translate into improved survival [11, 12]. In this patient the molecular findings were notable for biallelic TP53 mutations with different VAFs of 3% (clone A) and 6% (clone B) at baseline. After six months of therapy, while in hematologic remission, the larger clone (clone B) was reduced to < 1% in the bone marrow, whereas the smaller clone (clone A) expanded to a VAF of 28%, consistent with selection pressure exerted by therapy. Three months after treatment discontinuation, during ongoing stable hematologic remission, clone size in the peripheral blood remained stable, with clone A persisting at a VAF of 29%, while clone B remained < 1%.

In patient 6, the ETV6 c.602T > C variant was identified as a germline variant with a population frequency of 0.04% and was classified as benign/likely benign [13].

In patient 7, persistence of DNMT3A and TET2 mutations was interpreted as clonal hematopoiesis [14].

## Discussion

Although HMA–VEN represents the standard of care for the treatment of elderly patients with AML, the optimal venetoclax dosing schedule and the appropriate duration of therapy in patients who achieve CR remain to be defined. With respect to venetoclax, dosing must balance efficacy and toxicity. In particular, neutrophil counts are critical for treatment-associated risk, which is predominantly driven by infections [1, 15]. This risk is most pronounced during cycle 1, when residual normal hematopoiesis is maximally suppressed by malignant cells. Accordingly, venetoclax was discontinued after seven days in patients with neutrophil counts < 100/µL, whereas a 14-day schedule was applied in subsequent cycles. Recent studies have consistently demonstrated that shorter venetoclax exposure of 7 or 14 days is associated with comparable efficacy and reduced toxicity [9, 15, 16].

The option of treatment discontinuation in patients with CR represents another important aspect and has been addressed in several retrospective studies [3, 17–19]. Chua et al. [3] reported prolonged treatment-free remission after cessation of venetoclax-based therapy in elderly patients with AML in CR. In their analysis of 29 patients treated for at least 12 months, no difference in relapse risk or overall survival (OS) was observed between patients who electively discontinued therapy (*n* = 12; median treatment duration 19.3 months) and those who continued treatment until disease progression (*n* = 16; median treatment duration 28.8 months). Notably, outcomes in the treatment discontinuation group even tended to be superior.

Similarly, Garcia et al. [17] demonstrated in a retrospective cohort of 62 patients that those who discontinued therapy due to poor tolerance had outcomes comparable to patients who continued azacitidine monotherapy. Whether maintenance azacitidine in patients initially treated with HMA–VEN is as effective as in AML patients pretreated with aplasiogenic therapy, as suggested by Wei et al. [20], remains to be determined.

Importantly, patients who relapse after time-limited therapy may achieve a second remission upon retreatment with the same venetoclax-based regimen, as observed in patient 4 of our series and in two of three patients reported by Chua et al. [3]. For patients with FLT3-ITD, such as patient 4, gilteritinib represents an additional therapeutic option in the event of insufficient response or second relapse. At present, FLT3, IDH, and menin inhibitors are not approved for first-line therapy in Germany.

However, our case series also has limitations: while the sensitivity for detecting somatic mutations in our patients was limited to 1%, a sensitivity of 0.01% for MRD detection may be more reliable for guiding treatment discontinuation, as suggested by Gomez de Antonio et al. [19] and currently being prospectively evaluated in an ongoing trial [21].

Furthermore, one patient with MDS-EB was included in this case series and treated off-label with HMA/VEN, based on the expectation that higher CR rates with combination therapy might translate into improved survival [11, 12]. However, this benefit has not been confirmed in the randomized phase 3 VERONA trial, although subgroup analyses are ongoing [22]. Against this background, the inclusion of this patient is of interest but should be interpreted with caution.

Taken together, data from retrospective studies and the findings of our case series suggest that time-limited treatment with HMA–VEN in elderly AML patients who achieve CR is a feasible and clinically reasonable approach that warrants prospective evaluation.

## Supplementary information

Below is the link to the electronic supplementary material.

Supplementary Material 1

## Data Availability

No datasets were generated or analysed during the current study.
