# Supplementary material for: Fixed-time treatment of elderly patients with acute myeloid leukemia (AML) or high-risk myelodysplastic syndrome (MDS) using hypomethylating agents and venetoclax – a case series
Source: Ann Hematol. 2026 May 22;105(6):292. doi: 10.1007/s00277-026-07076-z (PMC13197376; doi:10.1007/s00277-026-07076-z)
Supplement: Supplementary file 1 — Supplementary Material 1 [file 277_2026_7076_MOESM1_ESM.docx]

Supplementary Material 1

Additional information on the NGS panel, including all targeted regions.

Additional information:

Bioinformatic processing included adapter trimming (cutadapt v5.2), alignment to the hg19 reference genome (bwa v0.7.17), and variant calling using SAMtools (v1.23) and SeqNext software (JSI, v5.4.3). Variants were reported according to HGVS nomenclature and classified following ACMG guidelines; only pathogenic or likely pathogenic variants (class 4–5) were included in the analysis. Sequencing achieved a mean coverage of 3451×, with >99% of target regions covered at ≥50× depth. The analytical sensitivity for variant detection was approximately 4% variant allele frequency (VAF). Copy number alterations in selected genes were additionally assessed by multiplex ligation-dependent probe amplification (MLPA) when indicated.

List targeted regions:

chr1 1718750 1718896 GNB1 NM_002074.5 exon 11

chr1 1720472 1720728 GNB1 NM_002074.5 exon 10

chr1 1721814 1722055 GNB1 NM_002074.5 exon 9

chr1 1724664 1724770 GNB1 NM_002074.5 exon 8

chr1 1735838 1736040 GNB1 NM_002074.5 exon 7

chr1 1737894 1737997 GNB1 NM_002074.5 exon 6

chr1 1747175 1747321 GNB1 NM_002074.5 exon 5

chr1 1749256 1749334 GNB1 NM_002074.5 exon 4

chr1 1756816 1756912 GNB1 NM_002074.5 exon 3

chr1 27022875 27024051 ARID1A NM_006015.6 exon 1

chr1 27056122 27056374 ARID1A NM_006015.6 exon 2

chr1 27057623 27058115 ARID1A NM_006015.6 exon 3

chr1 27059147 27059303 ARID1A NM_006015.6 exon 4

chr1 27087327 27087607 ARID1A NM_006015.6 exon 5

chr1 27087855 27087984 ARID1A NM_006015.6 exon 6

chr1 27088623 27088830 ARID1A NM_006015.6 exon 7

chr1 27089444 27089796 ARID1A NM_006015.6 exon 8

chr1 27092692 27092877 ARID1A NM_006015.6 exon 9

chr1 27092928 27093077 ARID1A NM_006015.6 exon 10

chr1 27094261 27094510 ARID1A NM_006015.6 exon 11

chr1 27097590 27097837 ARID1A NM_006015.6 exon 12

chr1 27098971 27099143 ARID1A NM_006015.6 exon 13

chr1 27099283 27099498 ARID1A NM_006015.6 exon 14

chr1 27099817 27100007 ARID1A NM_006015.6 exon 15

chr1 27100051 27100228 ARID1A NM_006015.6 exon 16

chr1 27100273 27100409 ARID1A NM_006015.6 exon 17

chr1 27100800 27101731 ARID1A NM_006015.6 exon 18

chr1 27102048 27102218 ARID1A NM_006015.6 exon 19

chr1 27105494 27107267 ARID1A NM_006015.6 exon 20

chr1 36931938 36932448 CSF3R NM_000760.4 exon 17

chr1 36932811 36932932 CSF3R NM_000760.4 exon 16

chr1 36933139 36933272 CSF3R NM_000760.4 exon 15

chr1 36933403 36933583 CSF3R NM_000760.4 exon 14

chr1 43803500 43803618 MPL NM_005373.3 exon 1

chr1 43803750 43803922 MPL NM_005373.3 exon 2

chr1 43804193 43804411 MPL NM_005373.3 exon 3

chr1 43804922 43805260 MPL NM_005373.3 exon 4

chr1 43805615 43805817 MPL NM_005373.3 exon 5

chr1 43806038 43806204 MPL NM_005373.3 exon 6

chr1 43812096 43812320 MPL NM_005373.3 exon 7

chr1 43812443 43812625 MPL NM_005373.3 exon 8

chr1 43814494 43814693 MPL NM_005373.3 exon 9

chr1 43814914 43815050 MPL NM_005373.3 exon 10

chr1 43817867 43817994 MPL NM_005373.3 exon 11

chr1 43818169 43818463 MPL NM_005373.3 exon 12

chr1 65300225 65300360 JAK1 NM_002227.4 exon 25

chr1 65301059 65301209 JAK1 NM_002227.4 exon 24

chr1 65301761 65301918 JAK1 NM_002227.4 exon 23

chr1 65303595 65303807 JAK1 NM_002227.4 exon 22

chr1 65304128 65304292 JAK1 NM_002227.4 exon 21

chr1 65305266 65305498 JAK1 NM_002227.4 exon 20

chr1 65306908 65307042 JAK1 NM_002227.4 exon 19

chr1 65307114 65307304 JAK1 NM_002227.4 exon 18

chr1 65309727 65309918 JAK1 NM_002227.4 exon 17

chr1 65310417 65310592 JAK1 NM_002227.4 exon 16

chr1 65311176 65311343 JAK1 NM_002227.4 exon 15

chr1 65312312 65312439 JAK1 NM_002227.4 exon 14

chr1 65313195 65313378 JAK1 NM_002227.4 exon 13

chr1 65316467 65316613 JAK1 NM_002227.4 exon 12

chr1 65321172 65321401 JAK1 NM_002227.4 exon 11

chr1 65323319 65323482 JAK1 NM_002227.4 exon 10

chr1 65325768 65325965 JAK1 NM_002227.4 exon 9

chr1 65330450 65330675 JAK1 NM_002227.4 exon 8

chr1 65332529 65332911 JAK1 NM_002227.4 exon 7

chr1 65334974 65335177 JAK1 NM_002227.4 exon 6

chr1 65339033 65339226 JAK1 NM_002227.4 exon 5

chr1 65344688 65344851 JAK1 NM_002227.4 exon 4

chr1 65348940 65349178 JAK1 NM_002227.4 exon 3

chr1 65351922 65351967 JAK1 NM_002227.4 exon 2

chr1 67861520 67861521 IL12RB2 NM_001559.2 rs2229546

chr1 115251136 115251295 NRAS NM_002524.5 exon 5

chr1 115252170 115252369 NRAS NM_002524.5 exon 4

chr1 115256401 115256619 NRAS NM_002524.5 exon 3

chr1 115258651 115258801 NRAS NM_002524.5 exon 2

chr1 179520506 179520507 NPHS2 NM_014625.2 rs1410592

chr2 25457128 25457309 DNMT3A NM_022552.5 exon 23

chr2 25458556 25458714 DNMT3A NM_022552.5 exon 22

chr2 25459785 25459894 DNMT3A NM_022552.5 exon 21

chr2 25461979 25462104 DNMT3A NM_022552.5 exon 20

chr2 25463151 25463339 DNMT3A NM_022552.5 exon 19

chr2 25463489 25463619 DNMT3A NM_022552.5 exon 18

chr2 25464411 25464596 DNMT3A NM_022552.5 exon 17

chr2 25466747 25466871 DNMT3A NM_022552.5 exon 16

chr2 25467004 25467227 DNMT3A NM_022552.5 exon 15

chr2 25467389 25467541 DNMT3A NM_022552.5 exon 14

chr2 25468102 25468221 DNMT3A NM_022552.5 exon 13

chr2 25468869 25468953 DNMT3A NM_022552.5 exon 12

chr2 25469009 25469198 DNMT3A NM_022552.5 exon 11

chr2 25469469 25469665 DNMT3A NM_022552.5 exon 10

chr2 25469900 25470047 DNMT3A NM_022552.5 exon 9

chr2 25470440 25470638 DNMT3A NM_022552.5 exon 8

chr2 25470886 25471141 DNMT3A NM_022552.5 exon 7

chr2 25497790 25497976 DNMT3A NM_022552.5 exon 6

chr2 25498349 25498432 DNMT3A NM_022552.5 exon 5

chr2 25505290 25505600 DNMT3A NM_022552.5 exon 4

chr2 25522988 25523132 DNMT3A NM_022552.5 exon 3

chr2 25536762 25536873 DNMT3A NM_022552.5 exon 2

chr2 25972545 25973302 ASXL2 NM_018263.6 exon 12

chr2 25976383 25976528 ASXL2 NM_018263.6 exon 11

chr2 169789016 169789017 ABCB11 NM_003742.2 rs497692

chr2 198265419 198265680 SF3B1 NM_012433.4 exon 18

chr2 198266446 198266632 SF3B1 NM_012433.4 exon 16

chr2 198266689 198266874 SF3B1 NM_012433.4 exon 15

chr2 198267260 198267570 SF3B1 NM_012433.4 exon 14

chr2 198267653 198267779 SF3B1 NM_012433.4 exon 13

chr2 209113073 209113404 IDH1 NM_005896.4 exon 4

chr2 227896976 227896977 COL4A4 NM_000092.4 rs10203363

chr3 4403767 4403768 SUMF1 NM_182760.3 rs2819561

chr3 105377794 105378093 CBLB NM_170662.5 exon 19

chr3 105389057 105389216 CBLB NM_170662.5 exon 18

chr3 105397255 105397435 CBLB NM_170662.5 exon 17

chr3 105400303 105400474 CBLB NM_170662.5 exon 16

chr3 105400548 105400682 CBLB NM_170662.5 exon 15

chr3 105404144 105404330 CBLB NM_170662.5 exon 14

chr3 105412318 105412452 CBLB NM_170662.5 exon 13

chr3 105420918 105421323 CBLB NM_170662.5 exon 12

chr3 105422812 105423037 CBLB NM_170662.5 exon 11

chr3 105438871 105439114 CBLB NM_170662.5 exon 10

chr3 105452833 105453004 CBLB NM_170662.5 exon 9

chr3 105455995 105456122 CBLB NM_170662.5 exon 8

chr3 105459318 105459495 CBLB NM_170662.5 exon 7

chr3 105464741 105464902 CBLB NM_170662.5 exon 6

chr3 105470286 105470482 CBLB NM_170662.5 exon 5

chr3 105495220 105495406 CBLB NM_170662.5 exon 4

chr3 105572238 105572528 CBLB NM_170662.5 exon 3

chr3 105586234 105586441 CBLB NM_170662.5 exon 2

chr3 128199842 128200181 GATA2 NM_032638.5 exon 6

chr3 128200642 128200807 GATA2 NM_032638.5 exon 5

chr3 128202683 128202868 GATA2 NM_032638.5 exon 4

chr3 128204550 128205231 GATA2 NM_032638.5 exon 3

chr3 128205626 128205894 GATA2 NM_032638.5 exon 2

chr3 136057076 136057139 STAG1 NM_005862.3 exon 34

chr3 136057192 136057312 STAG1 NM_005862.3 exon 33

chr3 136059313 136059467 STAG1 NM_005862.3 exon 32

chr3 136060263 136060413 STAG1 NM_005862.3 exon 31

chr3 136062654 136062868 STAG1 NM_005862.3 exon 30

chr3 136067980 136068225 STAG1 NM_005862.3 exon 29

chr3 136076542 136076710 STAG1 NM_005862.3 exon 28

chr3 136077970 136078158 STAG1 NM_005862.3 exon 27

chr3 136082188 136082329 STAG1 NM_005862.3 exon 26

chr3 136085765 136085944 STAG1 NM_005862.3 exon 25

chr3 136087930 136088144 STAG1 NM_005862.3 exon 24

chr3 136096482 136096614 STAG1 NM_005862.3 exon 23

chr3 136117571 136117691 STAG1 NM_005862.3 exon 22

chr3 136136707 136136834 STAG1 NM_005862.3 exon 21

chr3 136139915 136140025 STAG1 NM_005862.3 exon 20

chr3 136141232 136141475 STAG1 NM_005862.3 exon 19

chr3 136141590 136141719 STAG1 NM_005862.3 exon 18

chr3 136141774 136141906 STAG1 NM_005862.3 exon 17

chr3 136152378 136152521 STAG1 NM_005862.3 exon 16

chr3 136162109 136162266 STAG1 NM_005862.3 exon 15

chr3 136170855 136171009 STAG1 NM_005862.3 exon 14

chr3 136183703 136183850 STAG1 NM_005862.3 exon 13

chr3 136191235 136191354 STAG1 NM_005862.3 exon 12

chr3 136192361 136192499 STAG1 NM_005862.3 exon 11

chr3 136196111 136196274 STAG1 NM_005862.3 exon 10

chr3 136219045 136219158 STAG1 NM_005862.3 exon 9

chr3 136221450 136221641 STAG1 NM_005862.3 exon 8

chr3 136240035 136240279 STAG1 NM_005862.3 exon 7

chr3 136260941 136261057 STAG1 NM_005862.3 exon 6

chr3 136287587 136287723 STAG1 NM_005862.3 exon 5

chr3 136323131 136323335 STAG1 NM_005862.3 exon 4

chr3 136341968 136342110 STAG1 NM_005862.3 exon 3

chr3 136349692 136349760 STAG1 NM_005862.3 exon 2

chr3 184090281 184090986 THPO NM_000460.4 exon 6

chr3 184091183 184091390 THPO NM_000460.4 exon 5

chr3 184093283 184093409 THPO NM_000460.4 exon 4

chr3 184093656 184093823 THPO NM_000460.4 exon 3

chr3 184094015 184094067 THPO NM_000460.4 exon 2

chr4 5749904 5749905 EVC NM_153717.2 rs4688963

chr4 24572257 24572490 DHX15 NM_001358.3 exon 3

chr4 55140988 55141160 PDGFRA NM_006206.6 exon 12

chr4 55144043 55144193 PDGFRA NM_006206.6 exon 14

chr4 55151988 55152150 PDGFRA NM_006206.6 exon 18

chr4 55524162 55524268 KIT NM_000222.3 exon 1

chr4 55561658 55561967 KIT NM_000222.3 exon 2

chr4 55564430 55564751 KIT NM_000222.3 exon 3

chr4 55565776 55565952 KIT NM_000222.3 exon 4

chr4 55569870 55570078 KIT NM_000222.3 exon 5

chr4 55573244 55573473 KIT NM_000222.3 exon 6

chr4 55575570 55575725 KIT NM_000222.3 exon 7

chr4 55589730 55589884 KIT NM_000222.3 exon 8

chr4 55592003 55592236 KIT NM_000222.3 exon 9

chr4 55593364 55593510 KIT NM_000222.3 exon 10

chr4 55593562 55593728 KIT NM_000222.3 exon 11

chr4 55593969 55594113 KIT NM_000222.3 exon 12

chr4 55594157 55594307 KIT NM_000222.3 exon 13

chr4 55595481 55595671 KIT NM_000222.3 exon 14

chr4 55597474 55597605 KIT NM_000222.3 exon 15

chr4 55598017 55598184 KIT NM_000222.3 exon 16

chr4 55599216 55599378 KIT NM_000222.3 exon 17

chr4 55602644 55602795 KIT NM_000222.3 exon 18

chr4 55602867 55603006 KIT NM_000222.3 exon 19

chr4 55603321 55603466 KIT NM_000222.3 exon 20

chr4 55604575 55604743 KIT NM_000222.3 exon 21

chr4 57333782 57333930 SRP72 NM_006947.4 exon 1

chr4 57335799 57335959 SRP72 NM_006947.4 exon 2

chr4 57337866 57338029 SRP72 NM_006947.4 exon 3

chr4 57340200 57340383 SRP72 NM_006947.4 exon 4

chr4 57340426 57340577 SRP72 NM_006947.4 exon 5

chr4 57342817 57342888 SRP72 NM_006947.4 exon 6

chr4 57344525 57344689 SRP72 NM_006947.4 exon 7

chr4 57344738 57344835 SRP72 NM_006947.4 exon 8

chr4 57349285 57349456 SRP72 NM_006947.4 exon 9

chr4 57350882 57351050 SRP72 NM_006947.4 exon 10

chr4 57352471 57352583 SRP72 NM_006947.4 exon 11

chr4 57354095 57354199 SRP72 NM_006947.4 exon 12

chr4 57355534 57355669 SRP72 NM_006947.4 exon 13

chr4 57356479 57356622 SRP72 NM_006947.4 exon 14

chr4 57356714 57356831 SRP72 NM_006947.4 exon 15

chr4 57357577 57357754 SRP72 NM_006947.4 exon 16

chr4 57361503 57361580 SRP72 NM_006947.4 exon 17

chr4 57366682 57366881 SRP72 NM_006947.4 exon 18

chr4 57367830 57368047 SRP72 NM_006947.4 exon 19

chr4 106155080 106158528 TET2 NM_001127208.3 exon 3

chr4 106162476 106162606 TET2 NM_001127208.3 exon 4

chr4 106163971 106164104 TET2 NM_001127208.3 exon 5

chr4 106164707 106164955 TET2 NM_001127208.3 exon 6

chr4 106180756 106180946 TET2 NM_001127208.3 exon 7

chr4 106182896 106183025 TET2 NM_001127208.3 exon 8

chr4 106190747 106190924 TET2 NM_001127208.3 exon 9

chr4 106193701 106194095 TET2 NM_001127208.3 exon 10

chr4 106196185 106197696 TET2 NM_001127208.3 exon 11

chr5 82834630 82834631 VCAN NM_004385.4 rs309557

chr5 148884990 148885178 CSNK1A1 NM_001892.6 exon 9

chr5 148886570 148886716 CSNK1A1 NM_001892.6 exon 8

chr5 148889422 148889536 CSNK1A1 NM_001892.6 exon 7

chr5 148891337 148891455 CSNK1A1 NM_001892.6 exon 6

chr5 148892613 148892792 CSNK1A1 NM_001892.6 exon 5

chr5 148899833 148899971 CSNK1A1 NM_001892.6 exon 4

chr5 148904588 148904754 CSNK1A1 NM_001892.6 exon 3

chr5 170837511 170837589 NPM1 NM_002520.7 exon 11

chr5 176938772 176938948 DDX41 NM_016222.4 exon 17

chr5 176939077 176939227 DDX41 NM_016222.4 exon 16

chr5 176939303 176939414 DDX41 NM_016222.4 exon 15

chr5 176939477 176939666 DDX41 NM_016222.4 exon 14

chr5 176939761 176939897 DDX41 NM_016222.4 exon 13

chr5 176939992 176940103 DDX41 NM_016222.4 exon 12

chr5 176940334 176940505 DDX41 NM_016222.4 exon 11

chr5 176940666 176940868 DDX41 NM_016222.4 exon 10

chr5 176941682 176941858 DDX41 NM_016222.4 exon 9

chr5 176941897 176942090 DDX41 NM_016222.4 exon 8

chr5 176942167 176942279 DDX41 NM_016222.4 exon 7

chr5 176942666 176942842 DDX41 NM_016222.4 exon 6

chr5 176942910 176943010 DDX41 NM_016222.4 exon 5

chr5 176943100 176943214 DDX41 NM_016222.4 exon 4

chr5 176943269 176943468 DDX41 NM_016222.4 exon 3

chr5 176943706 176943856 DDX41 NM_016222.4 exon 2

chr5 176943900 176943966 DDX41 NM_016222.4 exon 1

chr6 146755140 146755141 GRM1 NM_001278067.1 rs2942

chr7 48450157 48450158 ABCA13 NM_152701.3 rs17548783

chr7 50358638 50358717 IKZF1 NM_006060.6 exon 2

chr7 50367214 50367373 IKZF1 NM_006060.6 exon 3

chr7 50444211 50444511 IKZF1 NM_006060.6 exon 4

chr7 50450218 50450425 IKZF1 NM_006060.6 exon 5

chr7 50455023 50455188 IKZF1 NM_006060.6 exon 6

chr7 50459407 50459581 IKZF1 NM_006060.6 exon 7

chr7 50467596 50468345 IKZF1 NM_006060.6 exon 8

chr7 92730621 92735430 SAMD9 NM_017654.4 exon 3

chr7 92760510 92765304 SAMD9L NM_152703.5 exon 5

chr7 101460900 101460969 CUX1 NM_181552.4 exon 1

chr7 101559375 101559525 CUX1 NM_181552.4 exon 2

chr7 101671358 101671445 CUX1 NM_181552.4 exon 3

chr7 101713599 101713717 CUX1 NM_181552.4 exon 4

chr7 101740624 101740801 CUX1 NM_181552.4 exon 5

chr7 101747596 101747759 CUX1 NM_181552.4 exon 6

chr7 101754958 101755074 CUX1 NM_181552.4 exon 7

chr7 101758467 101758573 CUX1 NM_181552.4 exon 8

chr7 101801820 101801908 CUX1 NM_181552.4 exon 9

chr7 101813706 101813850 CUX1 NM_181552.4 exon 10

chr7 101821729 101821957 CUX1 NM_181552.4 exon 11

chr7 101833073 101833171 CUX1 NM_181552.4 exon 12

chr7 101837102 101837190 CUX1 NM_181552.4 exon 13

chr7 101838767 101838903 CUX1 NM_181552.4 exon 14

chr7 101839894 101840605 CUX1 NM_181552.4 exon 15

chr7 101842062 101842167 CUX1 NM_181552.4 exon 16

chr7 101843331 101843472 CUX1 NM_181552.4 exon 17

chr7 101844620 101845504 CUX1 NM_181552.4 exon 18

chr7 101847651 101847856 CUX1 NM_181552.4 exon 19

chr7 101848374 101848470 CUX1 NM_181552.4 exon 20

chr7 101870627 101870969 CUX1 NM_181552.4 exon 21

chr7 101877312 101877540 CUX1 NM_181552.4 exon 22

chr7 101882580 101882884 CUX1 NM_181552.4 exon 23

chr7 101891672 101892342 CUX1 NM_181552.4 exon 24

chr7 140453055 140453213 BRAF NM_004333.6 exon 15

chr7 148504718 148504818 EZH2 NM_004456.5 exon 20

chr7 148506143 148506267 EZH2 NM_004456.5 exon 19

chr7 148506382 148506502 EZH2 NM_004456.5 exon 18

chr7 148507405 148507526 EZH2 NM_004456.5 exon 17

chr7 148508697 148508832 EZH2 NM_004456.5 exon 16

chr7 148511031 148511249 EZH2 NM_004456.5 exon 15

chr7 148511986 148512151 EZH2 NM_004456.5 exon 14

chr7 148512578 148512658 EZH2 NM_004456.5 exon 13

chr7 148513756 148513890 EZH2 NM_004456.5 exon 12

chr7 148514294 148514503 EZH2 NM_004456.5 exon 11

chr7 148514949 148515229 EZH2 NM_004456.5 exon 10

chr7 148516668 148516799 EZH2 NM_004456.5 exon 9

chr7 148523526 148523744 EZH2 NM_004456.5 exon 8

chr7 148524236 148524378 EZH2 NM_004456.5 exon 7

chr7 148525812 148525992 EZH2 NM_004456.5 exon 6

chr7 148526800 148526960 EZH2 NM_004456.5 exon 5

chr7 148529706 148529862 EZH2 NM_004456.5 exon 4

chr7 148543542 148543710 EZH2 NM_004456.5 exon 3

chr7 148544254 148544410 EZH2 NM_004456.5 exon 2

chr8 2796087 2796286 CSMD1 NM_033225.6 exon 70

chr8 2799974 2800146 CSMD1 NM_033225.6 exon 69

chr8 2806801 2806928 CSMD1 NM_033225.6 exon 68

chr8 2807733 2807885 CSMD1 NM_033225.6 exon 67

chr8 2808616 2808817 CSMD1 NM_033225.6 exon 66

chr8 2811726 2811810 CSMD1 NM_033225.6 exon 65

chr8 2813091 2813310 CSMD1 NM_033225.6 exon 64

chr8 2815198 2815349 CSMD1 NM_033225.6 exon 63

chr8 2818643 2818756 CSMD1 NM_033225.6 exon 62

chr8 2819968 2820181 CSMD1 NM_033225.6 exon 61

chr8 2820724 2820937 CSMD1 NM_033225.6 exon 60

chr8 2823277 2823496 CSMD1 NM_033225.6 exon 59

chr8 2824072 2824288 CSMD1 NM_033225.6 exon 58

chr8 2830619 2830841 CSMD1 NM_033225.6 exon 57

chr8 2831953 2832166 CSMD1 NM_033225.6 exon 56

chr8 2836114 2836342 CSMD1 NM_033225.6 exon 55

chr8 2855513 2855726 CSMD1 NM_033225.6 exon 54

chr8 2857460 2857673 CSMD1 NM_033225.6 exon 53

chr8 2875979 2876192 CSMD1 NM_033225.6 exon 52

chr8 2886821 2887055 CSMD1 NM_033225.6 exon 51

chr8 2909964 2910189 CSMD1 NM_033225.6 exon 50

chr8 2944599 2944827 CSMD1 NM_033225.6 exon 49

chr8 2949018 2949204 CSMD1 NM_033225.6 exon 48

chr8 2954351 2954579 CSMD1 NM_033225.6 exon 47

chr8 2964030 2964183 CSMD1 NM_033225.6 exon 46

chr8 2965220 2965340 CSMD1 NM_033225.6 exon 45

chr8 2966105 2966290 CSMD1 NM_033225.6 exon 44

chr8 2967660 2967877 CSMD1 NM_033225.6 exon 43

chr8 2975901 2976129 CSMD1 NM_033225.6 exon 42

chr8 2999967 3000216 CSMD1 NM_033225.6 exon 41

chr8 3008899 3009055 CSMD1 NM_033225.6 exon 40

chr8 3015399 3015508 CSMD1 NM_033225.6 exon 39

chr8 3019661 3019819 CSMD1 NM_033225.6 exon 38

chr8 3038612 3038756 CSMD1 NM_033225.6 exon 37

chr8 3045371 3045507 CSMD1 NM_033225.6 exon 36

chr8 3046389 3046553 CSMD1 NM_033225.6 exon 35

chr8 3047414 3047657 CSMD1 NM_033225.6 exon 34

chr8 3057216 3057351 CSMD1 NM_033225.6 exon 33

chr8 3059114 3059267 CSMD1 NM_033225.6 exon 32

chr8 3063006 3063162 CSMD1 NM_033225.6 exon 31

chr8 3071999 3072233 CSMD1 NM_033225.6 exon 30

chr8 3076757 3076984 CSMD1 NM_033225.6 exon 29

chr8 3081231 3081409 CSMD1 NM_033225.6 exon 28

chr8 3087542 3087773 CSMD1 NM_033225.6 exon 27

chr8 3141646 3141888 CSMD1 NM_033225.6 exon 26

chr8 3165197 3165363 CSMD1 NM_033225.6 exon 25

chr8 3165814 3166045 CSMD1 NM_033225.6 exon 24

chr8 3200796 3200992 CSMD1 NM_033225.6 exon 23

chr8 3205494 3205703 CSMD1 NM_033225.6 exon 22

chr8 3216654 3216882 CSMD1 NM_033225.6 exon 21

chr8 3224534 3224789 CSMD1 NM_033225.6 exon 20

chr8 3226756 3226912 CSMD1 NM_033225.6 exon 19

chr8 3244996 3245224 CSMD1 NM_033225.6 exon 18

chr8 3253696 3253923 CSMD1 NM_033225.6 exon 17

chr8 3256893 3257071 CSMD1 NM_033225.6 exon 16

chr8 3263529 3263763 CSMD1 NM_033225.6 exon 15

chr8 3265401 3265767 CSMD1 NM_033225.6 exon 14

chr8 3266925 3267147 CSMD1 NM_033225.6 exon 13

chr8 3326214 3326366 CSMD1 NM_033225.6 exon 12

chr8 3351125 3351268 CSMD1 NM_033225.6 exon 11

chr8 3432447 3432608 CSMD1 NM_033225.6 exon 10

chr8 3443638 3443802 CSMD1 NM_033225.6 exon 9

chr8 3474212 3474339 CSMD1 NM_033225.6 exon 8

chr8 3565916 3566033 CSMD1 NM_033225.6 exon 7

chr8 3611432 3611584 CSMD1 NM_033225.6 exon 6

chr8 3855405 3855652 CSMD1 NM_033225.6 exon 5

chr8 3889407 3889641 CSMD1 NM_033225.6 exon 4

chr8 4277455 4277607 CSMD1 NM_033225.6 exon 3

chr8 4494844 4495100 CSMD1 NM_033225.6 exon 2

chr8 4851834 4851958 CSMD1 NM_033225.6 exon 1

chr8 94935937 94935938 PDP1 NM_018444.3 rs4735258

chr8 117859719 117859950 RAD21 NM_006265.3 exon 14

chr8 117861165 117861288 RAD21 NM_006265.3 exon 13

chr8 117862837 117863026 RAD21 NM_006265.3 exon 12

chr8 117864167 117864355 RAD21 NM_006265.3 exon 11

chr8 117864768 117864967 RAD21 NM_006265.3 exon 10

chr8 117866464 117866727 RAD21 NM_006265.3 exon 9

chr8 117868385 117868547 RAD21 NM_006265.3 exon 8

chr8 117868865 117869030 RAD21 NM_006265.3 exon 7

chr8 117869486 117869732 RAD21 NM_006265.3 exon 6

chr8 117870571 117870717 RAD21 NM_006265.3 exon 5

chr8 117874060 117874199 RAD21 NM_006265.3 exon 4

chr8 117875349 117875518 RAD21 NM_006265.3 exon 3

chr8 117878805 117878988 RAD21 NM_006265.3 exon 2

chr9 5069905 5070072 JAK2 NM_004972.4 exon 12

chr9 5072472 5072646 JAK2 NM_004972.4 exon 13

chr9 5073678 5073805 JAK2 NM_004972.4 exon 14

chr9 5077433 5077600 JAK2 NM_004972.4 exon 15

chr9 5078286 5078464 JAK2 NM_004972.4 exon 16

chr9 100190780 100190781 TDRD7 NM_014290.2 rs1381532

chr9 133738130 133738442 ABL1 NM_007313.3 exon 4

chr9 133747496 133747620 ABL1 NM_007313.3 exon 5

chr9 133748227 133748444 ABL1 NM_007313.3 exon 6

chr9 133750235 133750459 ABL1 NM_007313.3 exon 7

chr9 133753782 133753974 ABL1 NM_007313.3 exon 8

chr9 133755435 133755564 ABL1 NM_007313.3 exon 9

chr9 133755867 133756071 ABL1 NM_007313.3 exon 10

chr9 133759336 133761090 ABL1 NM_007313.3 exon 11

chr10 27382594 27382748 ANKRD26 NM_014915.3 exon 2

chr10 27388994 27389275 ANKRD26 NM_014915.3 exon 1

chr10 89692750 89693028 PTEN NM_000314.8 exon 5

chr10 89717590 89717796 PTEN NM_000314.8 exon 7

chr10 100219314 100219315 HPSE2 NM_021828.4 rs10883099

chr11 532616 532775 HRAS NM_005343.4 exon 5

chr11 533433 533632 HRAS NM_005343.4 exon 4

chr11 533746 533964 HRAS NM_005343.4 exon 3

chr11 534192 534342 HRAS NM_005343.4 exon 2

chr11 16133413 16133414 SOX6 NM_033326.3 rs4617548

chr11 32413498 32413630 WT1 NM_024426.6 exon 9

chr11 32417783 32417973 WT1 NM_024426.6 exon 7

chr11 64533270 64533647 SF1 NM_004630.4 exon 13

chr11 64534352 64534571 SF1 NM_004630.4 exon 12

chr11 64534644 64534743 SF1 NM_004630.4 exon 11

chr11 64535023 64535336 SF1 NM_004630.4 exon 10

chr11 64535558 64535778 SF1 NM_004630.4 exon 9

chr11 64536474 64536621 SF1 NM_004630.4 exon 8

chr11 64536675 64536830 SF1 NM_004630.4 exon 7

chr11 64536878 64537101 SF1 NM_004630.4 exon 6

chr11 64537416 64537545 SF1 NM_004630.4 exon 5

chr11 64537708 64537900 SF1 NM_004630.4 exon 4

chr11 64540882 64540997 SF1 NM_004630.4 exon 3

chr11 64543950 64544118 SF1 NM_004630.4 exon 2

chr11 64545814 64545884 SF1 NM_004630.4 exon 1

chr11 118307208 118307679 KMT2A NM_001197104.2 exon 1

chr11 118339470 118339579 KMT2A NM_001197104.2 exon 2

chr11 118342357 118345050 KMT2A NM_001197104.2 exon 3

chr11 118347500 118347717 KMT2A NM_001197104.2 exon 4

chr11 118348662 118348936 KMT2A NM_001197104.2 exon 5

chr11 118350869 118350973 KMT2A NM_001197104.2 exon 6

chr11 118352410 118352827 KMT2A NM_001197104.2 exon 7

chr11 118353117 118353230 KMT2A NM_001197104.2 exon 8

chr11 118354878 118355049 KMT2A NM_001197104.2 exon 9

chr11 118355557 118355710 KMT2A NM_001197104.2 exon 10

chr11 118359309 118359495 KMT2A NM_001197104.2 exon 11

chr11 118360487 118360622 KMT2A NM_001197104.2 exon 12

chr11 118360824 118360984 KMT2A NM_001197104.2 exon 13

chr11 118361891 118362053 KMT2A NM_001197104.2 exon 14

chr11 118362439 118362663 KMT2A NM_001197104.2 exon 15

chr11 118363752 118363965 KMT2A NM_001197104.2 exon 16

chr11 118364983 118365133 KMT2A NM_001197104.2 exon 17

chr11 118365389 118365502 KMT2A NM_001197104.2 exon 18

chr11 118366395 118366628 KMT2A NM_001197104.2 exon 19

chr11 118366956 118367102 KMT2A NM_001197104.2 exon 20

chr11 118368631 118368808 KMT2A NM_001197104.2 exon 21

chr11 118369065 118369263 KMT2A NM_001197104.2 exon 22

chr11 118369998 118370155 KMT2A NM_001197104.2 exon 23

chr11 118370530 118370648 KMT2A NM_001197104.2 exon 24

chr11 118371682 118371882 KMT2A NM_001197104.2 exon 25

chr11 118372367 118372592 KMT2A NM_001197104.2 exon 26

chr11 118373093 118377381 KMT2A NM_001197104.2 exon 27

chr11 118378224 118378344 KMT2A NM_001197104.2 exon 28

chr11 118379831 118379935 KMT2A NM_001197104.2 exon 29

chr11 118380643 118380853 KMT2A NM_001197104.2 exon 30

chr11 118382646 118382760 KMT2A NM_001197104.2 exon 31

chr11 118390313 118390527 KMT2A NM_001197104.2 exon 32

chr11 118390652 118390799 KMT2A NM_001197104.2 exon 33

chr11 118391497 118391620 KMT2A NM_001197104.2 exon 34

chr11 118391983 118392152 KMT2A NM_001197104.2 exon 35

chr11 118392592 118392907 KMT2A NM_001197104.2 exon 36

chr11 119148856 119149027 CBL NM_005188.4 exon 8

chr11 119149200 119149443 CBL NM_005188.4 exon 9

chr12 993930 993931 WNK1 NM_018979.3 rs7300444

chr12 11803042 11803114 ETV6 NM_001987.5 exon 1

chr12 11905364 11905533 ETV6 NM_001987.5 exon 2

chr12 11992054 11992258 ETV6 NM_001987.5 exon 3

chr12 12006341 12006515 ETV6 NM_001987.5 exon 4

chr12 12022338 12022923 ETV6 NM_001987.5 exon 5

chr12 12037359 12037541 ETV6 NM_001987.5 exon 6

chr12 12038840 12038980 ETV6 NM_001987.5 exon 7

chr12 12043855 12044000 ETV6 NM_001987.5 exon 8

chr12 22778345 22778540 ETNK1 NM_018638.5 exon 1

chr12 22796677 22796976 ETNK1 NM_018638.5 exon 2

chr12 22811928 22812108 ETNK1 NM_018638.5 exon 3

chr12 22813977 22814159 ETNK1 NM_018638.5 exon 4

chr12 22824186 22824309 ETNK1 NM_018638.5 exon 5

chr12 22826414 22826614 ETNK1 NM_018638.5 exon 6

chr12 22837397 22837510 ETNK1 NM_018638.5 exon 7

chr12 22837796 22837908 ETNK1 NM_018638.5 exon 8

chr12 25362709 25362865 KRAS NM_004985.5 exon 5

chr12 25378528 25378727 KRAS NM_004985.5 exon 4

chr12 25380148 25380366 KRAS NM_004985.5 exon 3

chr12 25398188 25398338 KRAS NM_004985.5 exon 2

chr12 54686138 54687185 NFE2 NM_001261461.2 exon 4

chr12 54688899 54689052 NFE2 NM_001261461.2 exon 3

chr12 111855930 111856701 SH2B3 NM_005475.3 exon 2

chr12 112888102 112888336 PTPN11 NM_002834.5 exon 3

chr12 112926808 112926999 PTPN11 NM_002834.5 exon 13

chr13 28592584 28592746 FLT3 NM_004119.3 exon 20

chr13 28608004 28608371 FLT3 NM_004119.3 exon 14_15

chr13 39433606 39433607 FREM2 NM_207361.4 rs9532292

chr14 50769717 50769718 L2HGDH NM_024884.2 rs2297995

chr15 34528948 34528949 SLC12A6 NM_005135.2 rs4577050

chr15 90631799 90631999 IDH2 NM_002168.4 exon 4

chr16 70303580 70303581 AARS NM_001605.2 rs2070203

chr17 1556816 1556997 PRPF8 NM_006445.4 exon 39

chr17 1557051 1557330 PRPF8 NM_006445.4 exon 38

chr17 1558624 1558857 PRPF8 NM_006445.4 exon 37

chr17 1559666 1559879 PRPF8 NM_006445.4 exon 36

chr17 1559922 1560075 PRPF8 NM_006445.4 exon 35

chr17 1561527 1561695 PRPF8 NM_006445.4 exon 34

chr17 1561800 1562077 PRPF8 NM_006445.4 exon 33

chr17 1562631 1562862 PRPF8 NM_006445.4 exon 32

chr17 1563115 1563315 PRPF8 NM_006445.4 exon 31

chr17 1563706 1563892 PRPF8 NM_006445.4 exon 30

chr17 1563972 1564141 PRPF8 NM_006445.4 exon 29

chr17 1564267 1564476 PRPF8 NM_006445.4 exon 28

chr17 1564545 1564720 PRPF8 NM_006445.4 exon 27

chr17 1564885 1565104 PRPF8 NM_006445.4 exon 26

chr17 1565180 1565467 PRPF8 NM_006445.4 exon 25

chr17 1579779 1580025 PRPF8 NM_006445.4 exon 16

chr17 1580250 1580486 PRPF8 NM_006445.4 exon 15

chr17 1580839 1581008 PRPF8 NM_006445.4 exon 14

chr17 1581792 1581966 PRPF8 NM_006445.4 exon 13

chr17 1582036 1582195 PRPF8 NM_006445.4 exon 12

chr17 1582291 1582520 PRPF8 NM_006445.4 exon 11

chr17 1582565 1582724 PRPF8 NM_006445.4 exon 10

chr17 1582883 1583113 PRPF8 NM_006445.4 exon 9

chr17 1584000 1584145 PRPF8 NM_006445.4 exon 8

chr17 1584203 1584368 PRPF8 NM_006445.4 exon 7

chr17 1584752 1585004 PRPF8 NM_006445.4 exon 6

chr17 1585094 1585352 PRPF8 NM_006445.4 exon 5

chr17 1585403 1585607 PRPF8 NM_006445.4 exon 4

chr17 7572907 7573028 TP53 NM_000546.6 exon 11

chr17 7573907 7574053 TP53 NM_000546.6 exon 10

chr17 7576833 7576946 TP53 NM_000546.6 exon 9

chr17 7576999 7577175 TP53 NM_000546.6 exon 8

chr17 7577479 7577628 TP53 NM_000546.6 exon 7

chr17 7578157 7578309 TP53 NM_000546.6 exon 6

chr17 7578351 7578574 TP53 NM_000546.6 exon 5

chr17 7579292 7579610 TP53 NM_000546.6 exon 4

chr17 7579680 7579741 TP53 NM_000546.6 exon 3

chr17 7579819 7579932 TP53 NM_000546.6 exon 2

chr17 29422308 29422407 NF1 NM_001042492.3 exon 1

chr17 29482981 29483164 NF1 NM_001042492.3 exon 2

chr17 29486008 29486131 NF1 NM_001042492.3 exon 3

chr17 29490184 29490414 NF1 NM_001042492.3 exon 4

chr17 29496889 29497035 NF1 NM_001042492.3 exon 5

chr17 29508420 29508527 NF1 NM_001042492.3 exon 6

chr17 29508708 29508823 NF1 NM_001042492.3 exon 7

chr17 29509506 29509703 NF1 NM_001042492.3 exon 8

chr17 29527420 29527633 NF1 NM_001042492.3 exon 9

chr17 29528035 29528197 NF1 NM_001042492.3 exon 10

chr17 29528409 29528523 NF1 NM_001042492.3 exon 11

chr17 29533238 29533409 NF1 NM_001042492.3 exon 12

chr17 29541449 29541623 NF1 NM_001042492.3 exon 13

chr17 29546003 29546156 NF1 NM_001042492.3 exon 14

chr17 29548848 29548967 NF1 NM_001042492.3 exon 15

chr17 29550442 29550605 NF1 NM_001042492.3 exon 16

chr17 29552093 29552288 NF1 NM_001042492.3 exon 17

chr17 29553433 29553722 NF1 NM_001042492.3 exon 18

chr17 29554216 29554329 NF1 NM_001042492.3 exon 19

chr17 29554521 29554644 NF1 NM_001042492.3 exon 20

chr17 29556023 29556503 NF1 NM_001042492.3 exon 21

chr17 29556833 29557012 NF1 NM_001042492.3 exon 22

chr17 29557258 29557420 NF1 NM_001042492.3 exon 23

chr17 29557840 29557963 NF1 NM_001042492.3 exon 24

chr17 29559071 29559227 NF1 NM_001042492.3 exon 25

chr17 29559698 29559919 NF1 NM_001042492.3 exon 26

chr17 29560000 29560251 NF1 NM_001042492.3 exon 27

chr17 29562609 29562810 NF1 NM_001042492.3 exon 28

chr17 29562916 29563059 NF1 NM_001042492.3 exon 29

chr17 29575982 29576157 NF1 NM_001042492.3 exon 30

chr17 29579936 29580038 NF1 NM_001042492.3 exon 31

chr17 29585342 29585540 NF1 NM_001042492.3 exon 32

chr17 29586030 29586167 NF1 NM_001042492.3 exon 33

chr17 29587367 29587553 NF1 NM_001042492.3 exon 34

chr17 29588709 29588895 NF1 NM_001042492.3 exon 35

chr17 29592227 29592377 NF1 NM_001042492.3 exon 36

chr17 29652818 29653290 NF1 NM_001042492.3 exon 37

chr17 40354338 40354485 STAT5B NM_012448.4 exon 18

chr17 40354755 40354846 STAT5B NM_012448.4 exon 17

chr17 40359556 40359766 STAT5B NM_012448.4 exon 16

chr17 40362169 40362339 STAT5B NM_012448.4 exon 15

chr17 40474280 40474532 STAT3 NM_139276.3 exon 21

chr17 40498567 40498751 STAT3 NM_139276.3 exon 3

chr17 42284590 42284755 UBTF NM_014233.4 exon 21

chr17 42284802 42284985 UBTF NM_014233.4 exon 20

chr17 42285047 42285158 UBTF NM_014233.4 exon 19

chr17 42285212 42285299 UBTF NM_014233.4 exon 18

chr17 42286700 42286929 UBTF NM_014233.4 exon 17

chr17 42286993 42287121 UBTF NM_014233.4 exon 16

chr17 42287472 42287622 UBTF NM_014233.4 exon 15

chr17 42287666 42287861 UBTF NM_014233.4 exon 14

chr17 42288140 42288335 UBTF NM_014233.4 exon 13

chr17 42288387 42288540 UBTF NM_014233.4 exon 12

chr17 42288638 42288719 UBTF NM_014233.4 exon 11

chr17 42288954 42289135 UBTF NM_014233.4 exon 10

chr17 42289221 42289394 UBTF NM_014233.4 exon 9

chr17 42289692 42289842 UBTF NM_014233.4 exon 8

chr17 42290167 42290327 UBTF NM_014233.4 exon 7

chr17 42290566 42290670 UBTF NM_014233.4 exon 6

chr17 42293002 42293197 UBTF NM_014233.4 exon 5

chr17 42293254 42293377 UBTF NM_014233.4 exon 4

chr17 42293877 42294092 UBTF NM_014233.4 exon 3

chr17 42295520 42295617 UBTF NM_014233.4 exon 2

chr17 58677756 58678267 PPM1D NM_003620.4 exon 1

chr17 58700862 58701130 PPM1D NM_003620.4 exon 2

chr17 58711194 58711358 PPM1D NM_003620.4 exon 3

chr17 58725233 58725463 PPM1D NM_003620.4 exon 4

chr17 58733940 58734222 PPM1D NM_003620.4 exon 5

chr17 58740336 58740933 PPM1D NM_003620.4 exon 6

chr17 71197748 71197749 COG1 NM_018714.2 rs1037256

chr17 74732861 74733262 SRSF2 NM_003016.4 exon 1

chr18 21413869 21413870 LAMA3 NM_198129.1 rs9962023

chr18 42529826 42533325 SETBP1 NM_015559.3 exon 4

chr19 4047730 4048262 ZBTB7A NM_015898.4 exon 3

chr19 4053949 4055250 ZBTB7A NM_015898.4 exon 2

chr19 10267077 10267078 DNMT1 NM_001130823.1 rs2228611

chr19 13054507 13054747 CALR NM_004343.4 exon 9

chr19 17937532 17937739 JAK3 NM_000215.4 exon 24

chr19 17940897 17941047 JAK3 NM_000215.4 exon 23

chr19 17941292 17941449 JAK3 NM_000215.4 exon 22

chr19 17942017 17942229 JAK3 NM_000215.4 exon 21

chr19 17942463 17942627 JAK3 NM_000215.4 exon 20

chr19 17943308 17943537 JAK3 NM_000215.4 exon 19

chr19 17943579 17943758 JAK3 NM_000215.4 exon 18

chr19 17945360 17945550 JAK3 NM_000215.4 exon 17

chr19 17945641 17945832 JAK3 NM_000215.4 exon 16

chr19 17945872 17946044 JAK3 NM_000215.4 exon 15

chr19 17946713 17946880 JAK3 NM_000215.4 exon 14

chr19 17947918 17948042 JAK3 NM_000215.4 exon 13

chr19 17948721 17948892 JAK3 NM_000215.4 exon 12

chr19 17949052 17949219 JAK3 NM_000215.4 exon 11

chr19 17950266 17950492 JAK3 NM_000215.4 exon 10

chr19 17951019 17951170 JAK3 NM_000215.4 exon 9

chr19 17952178 17952375 JAK3 NM_000215.4 exon 8

chr19 17952429 17952591 JAK3 NM_000215.4 exon 7

chr19 17953105 17953439 JAK3 NM_000215.4 exon 6

chr19 17953816 17954001 JAK3 NM_000215.4 exon 5

chr19 17954169 17954320 JAK3 NM_000215.4 exon 4

chr19 17954566 17954729 JAK3 NM_000215.4 exon 3

chr19 17955023 17955246 JAK3 NM_000215.4 exon 2

chr19 33792224 33793340 CEBPA NM_004364.5 exon 1

chr19 45295620 45295791 CBLC NM_012116.4 exon 7

chr19 45296711 45296897 CBLC NM_012116.4 exon 8

chr19 49458166 49458239 BAX NM_138761.4 exon 1

chr19 49458785 49458876 BAX NM_138761.4 exon 2

chr19 49458924 49459110 BAX NM_138761.4 exon 3

chr19 49459435 49459610 BAX NM_138761.4 exon 4

chr19 49464047 49464191 BAX NM_138761.4 exon 5

chr19 49464769 49464913 BAX NM_138761.4 exon 6

chr19 56166451 56166539 U2AF2 NM_007279.3 exon 1

chr19 56170556 56170731 U2AF2 NM_007279.3 exon 2

chr19 56171523 56171607 U2AF2 NM_007279.3 exon 3

chr19 56171862 56172005 U2AF2 NM_007279.3 exon 4

chr19 56172384 56172575 U2AF2 NM_007279.3 exon 5

chr19 56173848 56174004 U2AF2 NM_007279.3 exon 6

chr19 56174952 56175130 U2AF2 NM_007279.3 exon 7

chr19 56179853 56179972 U2AF2 NM_007279.3 exon 8

chr19 56180016 56180178 U2AF2 NM_007279.3 exon 9

chr19 56180429 56180567 U2AF2 NM_007279.3 exon 10

chr19 56180790 56181078 U2AF2 NM_007279.3 exon 11

chr19 56185280 56185454 U2AF2 NM_007279.3 exon 12

chr20 6100088 6100089 FERMT1 NM_017671.4 rs10373

chr20 31022215 31025161 ASXL1 NM_015338.6 exon 13

chr20 57484385 57484498 GNAS NM_000516.7 exon 8

chr20 57484556 57484654 GNAS NM_000516.7 exon 9

chr21 36164412 36164927 RUNX1 NM_001754.5 exon 9

chr21 36171578 36171779 RUNX1 NM_001754.5 exon 8

chr21 36206687 36206918 RUNX1 NM_001754.5 exon 7

chr21 36231751 36231895 RUNX1 NM_001754.5 exon 6

chr21 36252834 36253030 RUNX1 NM_001754.5 exon 5

chr21 36259120 36259413 RUNX1 NM_001754.5 exon 4

chr21 36265202 36265280 RUNX1 NM_001754.5 exon 3

chr21 36421119 36421216 RUNX1 NM_001754.5 exon 2

chr21 44323590 44323591 NDUFV3 NM_021075.3 rs4148973

chr21 44514745 44514918 U2AF1 NM_006758.3 exon 6

chr21 44524405 44524532 U2AF1 NM_006758.3 exon 2

chr22 21141300 21141301 SERPIND1 NM_000185.3 rs4675

chr22 30730563 30730704 SF3A1 NM_005877.6 exon 16

chr22 30731436 30731547 SF3A1 NM_005877.6 exon 15

chr22 30731621 30731762 SF3A1 NM_005877.6 exon 14

chr22 30732995 30733189 SF3A1 NM_005877.6 exon 13

chr22 30733659 30733906 SF3A1 NM_005877.6 exon 12

chr22 30734758 30735043 SF3A1 NM_005877.6 exon 11

chr22 30735099 30735260 SF3A1 NM_005877.6 exon 10

chr22 30736165 30736390 SF3A1 NM_005877.6 exon 9

chr22 30736664 30736821 SF3A1 NM_005877.6 exon 8

chr22 30737661 30737894 SF3A1 NM_005877.6 exon 7

chr22 30738169 30738359 SF3A1 NM_005877.6 exon 6

chr22 30738774 30738888 SF3A1 NM_005877.6 exon 5

chr22 30740902 30741199 SF3A1 NM_005877.6 exon 4

chr22 30742281 30742528 SF3A1 NM_005877.6 exon 3

chr22 30748920 30749081 SF3A1 NM_005877.6 exon 2

chr22 30752699 30752801 SF3A1 NM_005877.6 exon 1

chrX 15339608 15339914 PIGA NM_002641.4 exon 6

chrX 15342767 15343013 PIGA NM_002641.4 exon 5

chrX 15343122 15343294 PIGA NM_002641.4 exon 4

chrX 15344016 15344188 PIGA NM_002641.4 exon 3

chrX 15349318 15350072 PIGA NM_002641.4 exon 2

chrX 15808599 15808679 ZRSR2 NM_005089.4 exon 1

chrX 15809037 15809156 ZRSR2 NM_005089.4 exon 2

chrX 15817975 15818096 ZRSR2 NM_005089.4 exon 3

chrX 15821791 15821939 ZRSR2 NM_005089.4 exon 4

chrX 15822214 15822340 ZRSR2 NM_005089.4 exon 5

chrX 15826336 15826414 ZRSR2 NM_005089.4 exon 6

chrX 15827303 15827461 ZRSR2 NM_005089.4 exon 7

chrX 15833780 15834033 ZRSR2 NM_005089.4 exon 8

chrX 15836690 15836785 ZRSR2 NM_005089.4 exon 9

chrX 15838310 15838459 ZRSR2 NM_005089.4 exon 10

chrX 15840834 15841385 ZRSR2 NM_005089.4 exon 11

chrX 39911342 39911673 BCOR NM_001123383.1 exon 15

chrX 39913119 39913315 BCOR NM_001123383.1 exon 14

chrX 39913489 39913606 BCOR NM_001123383.1 exon 13

chrX 39914601 39914786 BCOR NM_001123383.1 exon 12

chrX 39916388 39916594 BCOR NM_001123383.1 exon 11

chrX 39921372 39921666 BCOR NM_001123383.1 exon 10

chrX 39921979 39922344 BCOR NM_001123383.1 exon 9

chrX 39922841 39923123 BCOR NM_001123383.1 exon 8

chrX 39923569 39923872 BCOR NM_001123383.1 exon 7

chrX 39930206 39930432 BCOR NM_001123383.1 exon 6

chrX 39930870 39930963 BCOR NM_001123383.1 exon 5

chrX 39931582 39934453 BCOR NM_001123383.1 exon 4

chrX 39935687 39935805 BCOR NM_001123383.1 exon 3

chrX 39937077 39937202 BCOR NM_001123383.1 exon 2

chrX 44732778 44732978 KDM6A NM_021140.4 exon 1

chrX 44733150 44733253 KDM6A NM_021140.4 exon 2

chrX 44820509 44820657 KDM6A NM_021140.4 exon 3

chrX 44833891 44833980 KDM6A NM_021140.4 exon 4

chrX 44870186 44870284 KDM6A NM_021140.4 exon 5

chrX 44879835 44879995 KDM6A NM_021140.4 exon 6

chrX 44894156 44894250 KDM6A NM_021140.4 exon 7

chrX 44896880 44896954 KDM6A NM_021140.4 exon 8

chrX 44910934 44911067 KDM6A NM_021140.4 exon 9

chrX 44913054 44913220 KDM6A NM_021140.4 exon 10

chrX 44918231 44918369 KDM6A NM_021140.4 exon 11

chrX 44918472 44918731 KDM6A NM_021140.4 exon 12

chrX 44919247 44919421 KDM6A NM_021140.4 exon 13

chrX 44920549 44920684 KDM6A NM_021140.4 exon 14

chrX 44921872 44922013 KDM6A NM_021140.4 exon 15

chrX 44922647 44923082 KDM6A NM_021140.4 exon 16

chrX 44928804 44929622 KDM6A NM_021140.4 exon 17

chrX 44935922 44936091 KDM6A NM_021140.4 exon 18

chrX 44937625 44937770 KDM6A NM_021140.4 exon 19

chrX 44938371 44938616 KDM6A NM_021140.4 exon 20

chrX 44941801 44941905 KDM6A NM_021140.4 exon 21

chrX 44941940 44942054 KDM6A NM_021140.4 exon 22

chrX 44942685 44942873 KDM6A NM_021140.4 exon 23

chrX 44945090 44945244 KDM6A NM_021140.4 exon 24

chrX 44948968 44949195 KDM6A NM_021140.4 exon 25

chrX 44949948 44950129 KDM6A NM_021140.4 exon 26

chrX 44966635 44966801 KDM6A NM_021140.4 exon 27

chrX 44969304 44969514 KDM6A NM_021140.4 exon 28

chrX 44970607 44970676 KDM6A NM_021140.4 exon 29

chrX 47058182 47058338 UBA1 NM_003334.4 exon 2

chrX 47058427 47058525 UBA1 NM_003334.4 exon 3

chrX 47058588 47058796 UBA1 NM_003334.4 exon 4

chrX 47058859 47059033 UBA1 NM_003334.4 exon 5

chrX 47060273 47060419 UBA1 NM_003334.4 exon 6

chrX 47060655 47060785 UBA1 NM_003334.4 exon 7

chrX 47060857 47061029 UBA1 NM_003334.4 exon 8

chrX 47061535 47061672 UBA1 NM_003334.4 exon 9

chrX 47061737 47061923 UBA1 NM_003334.4 exon 10

chrX 47062017 47062233 UBA1 NM_003334.4 exon 11

chrX 47062322 47062466 UBA1 NM_003334.4 exon 12

chrX 47062513 47062633 UBA1 NM_003334.4 exon 13

chrX 47062920 47063115 UBA1 NM_003334.4 exon 14

chrX 47065327 47065532 UBA1 NM_003334.4 exon 15

chrX 47065627 47065863 UBA1 NM_003334.4 exon 16

chrX 48649497 48649756 GATA1 NM_002049.4 exon 2

chrX 48650231 48650648 GATA1 NM_002049.4 exon 3

chrX 48650710 48650895 GATA1 NM_002049.4 exon 4

chrX 48651559 48651724 GATA1 NM_002049.4 exon 5

chrX 48652180 48652591 GATA1 NM_002049.4 exon 6

chrX 55035593 55035796 ALAS2 NM_000032.5 exon 11

chrX 55039899 55040101 ALAS2 NM_000032.5 exon 10

chrX 55041160 55041468 ALAS2 NM_000032.5 exon 9

chrX 55041991 55042195 ALAS2 NM_000032.5 exon 8

chrX 55043899 55044118 ALAS2 NM_000032.5 exon 7

chrX 55046733 55046957 ALAS2 NM_000032.5 exon 6

chrX 55047465 55047727 ALAS2 NM_000032.5 exon 5

chrX 55050170 55050320 ALAS2 NM_000032.5 exon 4

chrX 55051131 55051293 ALAS2 NM_000032.5 exon 3

chrX 55052233 55052453 ALAS2 NM_000032.5 exon 2

chrX 76763809 76764127 ATRX NM_000489.6 exon 35

chrX 76776246 76776414 ATRX NM_000489.6 exon 34

chrX 76776861 76776996 ATRX NM_000489.6 exon 33

chrX 76777721 76777886 ATRX NM_000489.6 exon 32

chrX 76778710 76778899 ATRX NM_000489.6 exon 31

chrX 76812902 76813136 ATRX NM_000489.6 exon 30

chrX 76814120 76814337 ATRX NM_000489.6 exon 29

chrX 76829695 76829843 ATRX NM_000489.6 exon 28

chrX 76845284 76845430 ATRX NM_000489.6 exon 27

chrX 76849146 76849339 ATRX NM_000489.6 exon 26

chrX 76854860 76855069 ATRX NM_000489.6 exon 25

chrX 76855181 76855309 ATRX NM_000489.6 exon 24

chrX 76855883 76856053 ATRX NM_000489.6 exon 23

chrX 76872061 76872218 ATRX NM_000489.6 exon 22

chrX 76874254 76874469 ATRX NM_000489.6 exon 21

chrX 76875843 76876020 ATRX NM_000489.6 exon 20

chrX 76888675 76888892 ATRX NM_000489.6 exon 19

chrX 76889034 76889220 ATRX NM_000489.6 exon 18

chrX 76890065 76890214 ATRX NM_000489.6 exon 17

chrX 76931701 76931813 ATRX NM_000489.6 exon 10

chrX 76936992 76940105 ATRX NM_000489.6 exon 9

chrX 76940411 76940518 ATRX NM_000489.6 exon 8

chrX 123156458 123156541 STAG2 NM_001042749.2 exon 3

chrX 123159670 123159788 STAG2 NM_001042749.2 exon 4

chrX 123164791 123164995 STAG2 NM_001042749.2 exon 5

chrX 123171357 123171493 STAG2 NM_001042749.2 exon 6

chrX 123176399 123176515 STAG2 NM_001042749.2 exon 7

chrX 123178994 123179238 STAG2 NM_001042749.2 exon 8

chrX 123181184 123181375 STAG2 NM_001042749.2 exon 9

chrX 123182835 123182948 STAG2 NM_001042749.2 exon 10

chrX 123184016 123184179 STAG2 NM_001042749.2 exon 11

chrX 123184951 123185089 STAG2 NM_001042749.2 exon 12

chrX 123185145 123185264 STAG2 NM_001042749.2 exon 13

chrX 123189958 123190105 STAG2 NM_001042749.2 exon 14

chrX 123191696 123191847 STAG2 NM_001042749.2 exon 15

chrX 123195054 123195211 STAG2 NM_001042749.2 exon 16

chrX 123195601 123195744 STAG2 NM_001042749.2 exon 17

chrX 123196732 123196864 STAG2 NM_001042749.2 exon 18

chrX 123196946 123197075 STAG2 NM_001042749.2 exon 19

chrX 123197678 123197921 STAG2 NM_001042749.2 exon 20

chrX 123199706 123199816 STAG2 NM_001042749.2 exon 21

chrX 123200005 123200132 STAG2 NM_001042749.2 exon 22

chrX 123200186 123200306 STAG2 NM_001042749.2 exon 23

chrX 123202394 123202526 STAG2 NM_001042749.2 exon 24

chrX 123204979 123205193 STAG2 NM_001042749.2 exon 25

chrX 123210162 123210341 STAG2 NM_001042749.2 exon 26

chrX 123211787 123211928 STAG2 NM_001042749.2 exon 27

chrX 123215210 123215398 STAG2 NM_001042749.2 exon 28

chrX 123217251 123217419 STAG2 NM_001042749.2 exon 29

chrX 123220377 123220640 STAG2 NM_001042749.2 exon 30

chrX 123224405 123224634 STAG2 NM_001042749.2 exon 31

chrX 123224684 123224834 STAG2 NM_001042749.2 exon 32

chrX 123227848 123228014 STAG2 NM_001042749.2 exon 33

chrX 123229202 123229319 STAG2 NM_001042749.2 exon 34

chrX 123234404 123234467 STAG2 NM_001042749.2 exon 35

chrX 129139188 129139313 BCORL1 NM_021946.5 exon 2

chrX 129146534 129146664 BCORL1 NM_021946.5 exon 3

chrX 129146906 129150209 BCORL1 NM_021946.5 exon 4

chrX 129154940 129155145 BCORL1 NM_021946.5 exon 5

chrX 129156852 129156972 BCORL1 NM_021946.5 exon 6

chrX 129158945 129159374 BCORL1 NM_021946.5 exon 7

chrX 129162590 129162856 BCORL1 NM_021946.5 exon 8

chrX 129171322 129171528 BCORL1 NM_021946.5 exon 9

chrX 129173092 129173277 BCORL1 NM_021946.5 exon 10

chrX 129184672 129184789 BCORL1 NM_021946.5 exon 11

chrX 129185815 129186011 BCORL1 NM_021946.5 exon 12

chrX 129189809 129190131 BCORL1 NM_021946.5 exon 13

chrX 133511628 133511805 PHF6 NM_032335.3 exon 2

chrX 133511628 133511805 PHF6 NM_032458.3 exon 2

chrX 133512015 133512156 PHF6 NM_032335.3 exon 3

chrX 133512015 133512156 PHF6 NM_032458.3 exon 3

chrX 133527511 133527684 PHF6 NM_032335.3 exon 4

chrX 133527511 133527684 PHF6 NM_032458.3 exon 4

chrX 133527919 133528002 PHF6 NM_032335.3 exon 5

chrX 133527919 133528002 PHF6 NM_032458.3 exon 5

chrX 133547498 133547707 PHF6 NM_032335.3 exon 6

chrX 133547501 133547707 PHF6 NM_032458.3 exon 6

chrX 133547833 133548016 PHF6 NM_032335.3 exon 7

chrX 133547833 133548016 PHF6 NM_032458.3 exon 7

chrX 133549026 133549170 PHF6 NM_032458.3 exon 8

chrX 133549026 133549272 PHF6 NM_032335.3 exon 8

chrX 133551179 133551352 PHF6 NM_032458.3 exon 9

chrX 133559211 133559380 PHF6 NM_032458.3 exon 10
